# Concrete Repair Durability

**DOI:** 10.3390/ma13204535

**Published:** 2020-10-13

**Authors:** Lech Czarnecki, Robert Geryło, Krzysztof Kuczyński

**Affiliations:** Building Research Institute, ITB, Filtrowa 1, 00-611 Warsaw, Poland; r.gerylo@itb.pl (R.G.); k.kuczynski@itb.pl (K.K.)

**Keywords:** concrete repair durability, structure durability, durability determinants, repair durability distribution function, durability estimation, repair cycles, durability management strategy, large-scale durability development

## Abstract

The repairs of building structures are inevitable and indispensable. Repairs are used to restore or maintain the usability of existing facilities, often contributing to the extension of their expected service life, increasing the sustainability of building resources. Given that conservation rules are observed, repairs are also used to save monuments. The concept of repair durability brings to the foreground the durability of the repaired structure (after repair): what service life has been obtained/recovered as a result of the repair. Based on the available data (limited set), a generalised distribution function of repair durability was developed, with a disappointing course. This, however, applies (necessarily) to the past. Significant progress was shown to have been achieved in the theoretical and technical fundamentals of technical repair measures. In this situation, a prognostic distribution function was also designed for future repairs according to EN 1504. A rule of thumb called estimating concrete repair durability, CRD was proposed. The risk associated with estimating the durability of repairs was indicated. A reason for optimism is that proactive monitoring of the condition of the structure and, consequently, management of the repair strategy allows to reach the designed life of the structure.

## 1. Introduction

Durability is the building structure’s ability to remain in a functional condition, expressed in time units. Repair means eliminating defects, while a defect is a condition which requires interference. The conviction that concrete structures are durable is common and justified. Concrete structures are designed to provide at least 50 years of service, while public and monumental buildings are intended to last even longer. The service life of concrete structures is defined in “EN 206: Concrete—Requirements, Properties, Production and Compliance” [1] as a period in which the condition of concrete in the structure corresponds to the operating requirements [2,3]. The need for repair somehow (apparently) contradicts the conviction that concrete is durable. Various manuals on ensuring concrete durability and the design of durable structures often end with a chapter on “How to make repairs” [4]. The apparent contradiction is also evidenced by the fact that repairs—depending on circumstances—are intended to maintain or restore a building structure’s durability.

The purpose of the paper is to present the possibilities of estimating concrete repair durability, CRD in reference to the state of knowledge and engineering, and to indicate how a designed service life can be ensured through proactive management of the repair strategy. Historical information has been shown to be key to understanding several issues that the “technological” engineering approach leaves unresolved, and science is the last aid in saving some valuable buildings. In the paper, we focus particularly on the question of whether, in the current state-of-the-art, we can assess how many years, as a result of repairs, we will extend the life of the structure.

The inevitability of repairs is subject to the random nature of impacts on the structure, including: hitting, overloading, explosion etc., as well as extraordinary weather impacts (gusts of wind, extreme precipitation). These are unique and sudden instances. The inevitability of repairs is also determined thermodynamically, by the structure striving to achieve the condition of energy minimum and entropy increase, which can be confirmed by different corrosion processes, which are aggravated in certain conditions (chemically aggressive environment, abrasion etc.). Past experiences also indicate the indispensability of repairs. Six out of the seven wonders of the ancient world, i.e., the Hanging Gardens of Babylon (600 BCE), the Temple of Artemis at Ephesus (550 BCE), the Statue of Zeus at Olympia (435 BCE), the Mausoleum of Halicarnassus (352 BCE), the Colossus of Rhodes (281 BCE) and the Lighthouse of Alexandria (285 BCE) were severely damaged as a result of earthquakes and fires [5]. The destruction of these landmarks of civilisation can be mostly attributed to the lack of repairs.

**Figure 1:**
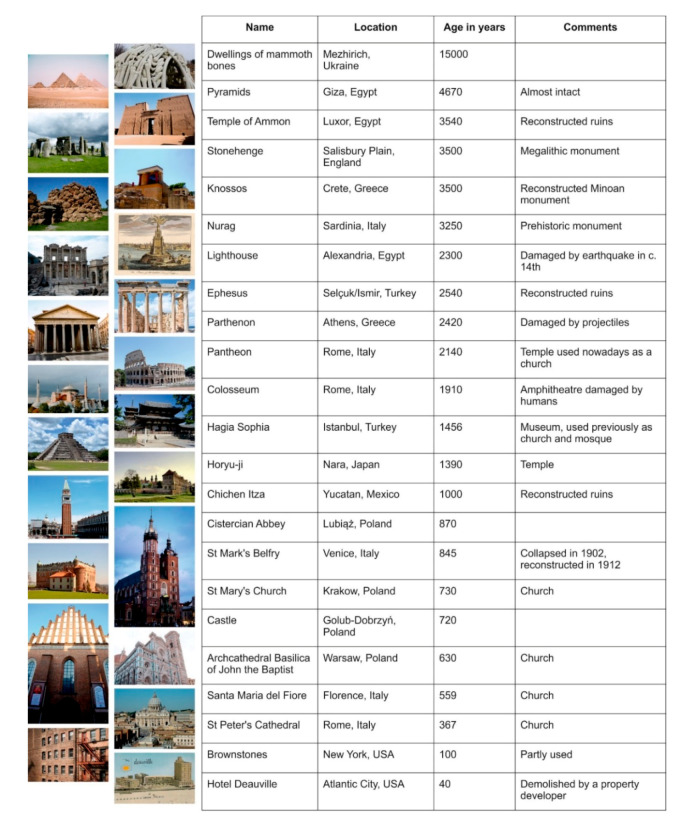
Selected monuments and their age [6], photo sources: unsplash.com, pxhere.com, pixabay.com, flickr.com, Wikipedia.com.

**Figure 2:**
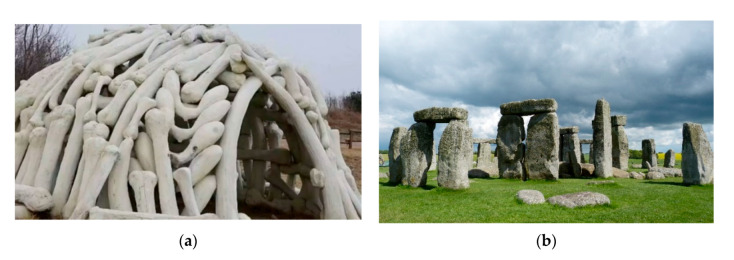
Examples of nearly intact structures dating back to 15,000 years ago: (**a**) dwellings of mammoth bones [source: arthistory390, Flickr.com], (**b**) megalithic monument [source: pxhere.com].

Among twenty monuments aged from several thousand to several dozen years (Figure 1) all but two were repaired, one by dismantling, which is also a repair method. Two of the structures that have lasted until our times are the megalithic circles of Stonehenge (3500 years old), England and the dwellings of mammoth bones (15,000 years old) in Mezhirich, Ukraine [5]. It can be observed that these two “everlasting” structures differ from other objects by the uniformity of their building material and the lack of construction joints (Figure 2).

Repairs are an inevitable and indispensable necessity. Given that conservation rules are observed, repairs are also used to save monuments, but first and foremost they help to restore or maintain the usability of existing structures. Consequently, repairs restore or extend expected durability. Due to the fact, that stone/brick skeletons have a much longer tradition in construction than that of Portland cement concrete, the historical approach to structural repairs is also much deeper for masonry structures.

In this context, it is worth recalling the very significant history of St. Peter’s dome repairs [7]. In 1742, after two centuries after its completion, the masonry dome, overloaded by its own weight showed several cracks. Alarmed, Pope Benedict XIV appointed a committee of scientists known as the Three Mathematicians, consisting of T. Le Seur, F. Jacquier and R.J. Boscovich to report the dome’s condition and ensure its restoration. It was an obvious sign that science was the last resource to save St. Peter’s Dome [8]. According to a report by the Three Mathematicians, the dome was in very dangerous condition and needed invasive intervention to ultimately change the architecture of the monument. Many other scholars found the dome quite safe. In light of the controversy, Benedict XIV consulted another famous Italian scholar G. Poleni [9]. In contrary to the Three Mathematicians, G. Poleni assured that the dome was still safe, despite its defects in construction and the use of poor masonry. Finally, successful repairs were carried out with the invaluable assistance of L. Vanritelli—an Italian engineer, the main architect of the basilica, a true practitioner of his craft. Recently (2019) the whole story has been refreshed and analysed by M. Como [10] and prior to that (1999, 2003) R. Mainstone [11,12] conducted extensive research. This certainly applies not only to sacral buildings, but also to industrial facilities, e.g., brick chimneys [13].

Many valuable lessons can be learned from these studies, which also apply to our topic. They can be defined as follows:

- Overall, in construction, the linkage between theory and practice is of great importance.
- In the field of repair, it may happen at any moment that practice triumphs over modern theory. It is sufficient to say that concrete repair has been performed since the introduction of concrete into construction practice (Eddystone lighthouse, 1756), but the standard for concrete repair was established in USA through the ACI-1999 Concrete Repair Manual, and in Europe in 2004–2013 according to the volumes of European Standards EN 1504.1-10.

Under some conditions, the repairs are also intended to increase the sustainability of building resources [14]. Furthermore, repairs tend to be treated as “the ultimate act of sustainability” [15]. In light of the above, the question about durability gains special meaning.

## 2. Question about Repair Durability

Authority in the field of concrete A.M. Neville in 1987 asked the question: Why do we have problems with the durability of concrete? [15]. Apart from the great progress in Portland cement and concrete technology, this is still a hot problem [16]. The question of a concrete repair durability is even more complicated [17,18].

The postulate of effective repair entails the requirement of repair durability. Data related to practice in this area are scarce. A building structure repair—contrary to its construction—entails certain discretion, arising from the professional embarrassment experienced in reference to the non-fulfilment of the common conviction of durability. The unwillingness to reveal information about the need to make repairs is additionally aggravated by the fact that 90% of the causes of repairs are workmanship and design errors (wrong choice of repair materials predominates among other causes) [19]. “A repair of a repair” is highly frustrating for those involved, and for a contracting company it can mean a disaster. Some unsuccessful repairs, e.g., using injection methods to repair cracks, cannot be undone. The title question about durability can be understood in two ways: as a question about the durability of the repair “as such” and the durability of the repaired structure (the structure after the repair). The difference is not clear by definition, but it is intuitively evident. Repairs tend to be compared to treating a patient (also in a semantic aspect) [20]. This would then mean the difference between a “successful operation” and the “patient’s post-surgical condition”. If a repair is successful, which is confirmed by a reasonably long life, the two terms are identical. The answer concerning the durability of repair as such in years [21] is relatively easier, but also rare. Normally, it applies to repairing a damage caused as a result of a single occurrence (see Section 5). A major renovation can be required due to the building structure’s general condition resulting from a number of destructive processes, such as: abrasive wear, erosion, frost corrosion, chemical corrosion of concrete or electrochemical corrosion of reinforcement, which are continuous, and their destructive effects accumulate, causing structural wear [22]. Then, the repair involves a number of methods, including injection of cracks, filling, repair and protection of reinforcement, and protection of the concrete surface. In such a case, a clear answer as to whether the repair was successful or unsuccessful is much more difficult. Many destructive processes cannot be eliminated completely but their progress can be hindered. Moreover, if compatibility requirements [23,24] are not met, new hazard sources can be introduced. For instance, if the concrete used for filling the defects is too tight, it can lead to the formation of concentration cells and progressive pitting at the repaired concrete contact points [18].

Let us illustrate these deliberations with an example: the Prince Józef Poniatowski overpass in Warsaw. In 1985, a major repair started on the Prince Józef Poniatowski overpass in Warsaw. It was the first time injection of cracks in concrete with epoxy resin and the repair of vast vertical areas by concrete spraying had been used. The repair took five years with 30 years having lapsed since. It can be concluded that if the repair rules are observed [4,25,26], the repair restores the usability condition, including load capacity, stability and safety, as well as the appearance of the repaired structure. The rule of thumb is to carry out the repair “based on causes” and not “based on symptoms”. If so, in the case of repairs caused by a random event, it means that the cause was removed and the conclusion can be drawn that the originally designed durability (service life) was restored. If the repair was made as a result of destruction due to long-lasting chemical, mechanical and/or electrochemical corrosion, such a conclusion carries a greater risk. A distinguished British scientist, prof. Jacques Heyman in his work “The stone skeleton” [27] (1995) formulated the following rule of thumb in reference to masonry structures: ‘If a structure stands for 5 min after being constructed, it will last for five hundred years’. In light of the current state of knowledge and technology, an attempt to paraphrase the rule in reference to concrete structure repairs seems premature. It would have to be ruled by strict conditions and be more limited (see Section 5). One should note that the set of experiences related to repaired concrete structures is smaller than the one covering masonry structures, both for the number of structures and for repairing traditions.

## 3. Repair as a Strategy of Managing Structure Durability

The difficulty in determining “repair durability” can be attributed to the interchangeable use of the terms “repair as restoring a structure’s use potential” (correct) and “repair as restoring a structure’s durability” (excess).

**Figure 3:**
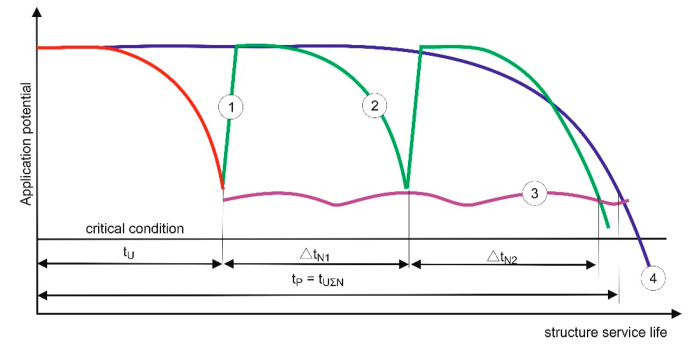
Typical repair cycles during the service life of a building structure subjected to degradation according to EN 2504-9 [28]: 1,2—repairs; 3—minimum condition of use; t_U_—original service life; Δt_N1_, Δt_N2_—service life gains as a result of repairs; t_P_—designed service life being a total of the original service life and service life gains as a result of subsequent repairs; 4—designed course of changes in the use potential.

**Figure 4:**
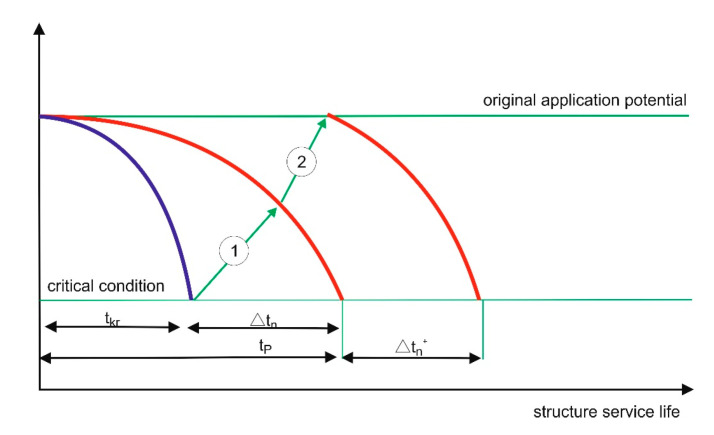
Schematic presentation of a repair cycle conducted as a result of a building structure’s premature degradation, e.g., due to impact. 1—repair restoring the use potential before damage; 2—repair restoring the original use potential; t_kr_—service life until damage; t_p_—designed service life; Δt_n—_service life regained owing to repair (restoring the designed service life); Δt_n_^+^_—_additional service life gained owing to restoring the original use potential.

The term “durable repair” is a mental shortcut, which means ensuring the durability of repair effects. Durability (service life) of the repaired building structure is important (rather than durability of the “repair”), which results from the influence of the use potential and the rate of destruction processes (Figure 3). A repair increases the use potential (restores the original use potential) and can reduce the rate of destruction processes, e.g., if the reinforcement lagging was replaced/improved and/or surface protective coating was applied (Figure 4).

**Figure 5:**
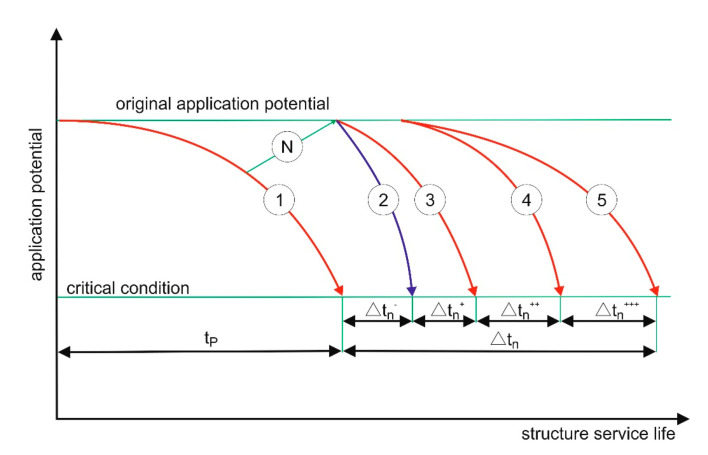
Schematic presentation of a repair cycle resulting in restoring the use potential: N—repair restoring the original use potential; 1—change in the use potential according to the design model; 2—change in the use potential after the repair, increased degradation rate as a result of new degradation sources triggered by the repair, e.g., electrochemical corrosion; 3—change in the use potential after the repair, unchanged degradation rate; 4—change in the use potential after the repair, degradation rate reduced e.g., as a result of lagging replacement; 5—change in the use potential after the repair, degradation rate additionally reduced by applying a surface protecting coating; tp—designed service life; Δt_n_^-^—additional service life gained owing to successful repair, reduced as a result of new degradation sources introduced by the repair; Δt_n_^+^—additional service life gained owing to successful repair, degradation rate unchanged; Δt_n_^++^—additional service life gained owing to successful repair, degradation rate reduced e.g., as a result of lagging replacement; Δt_n_^+++^—additional service life gained owing to successful repair, degradation rate additionally reduced by applying a surface protecting coating.

If premature degradation of a structure occurs, e.g., as a result of a random event or continuous destructive processes of significant intensity, EN 1504-9 [27] states that the designed service life can be restored in one complex operation (usually including different repair methods) or by performing simple operations regularly. The typical repair cycles presented in the standard, referring to the service life of a building structure subjected to degradation (Figure 5), are somewhat idealised and simplified:

- the use potential is always restored back to the original condition
- the destruction rate does not change as a result of the repair
- the fact that the repair occurs in time is not taken into account (the repair arrows are oriented upwards)

**Figure 6:**
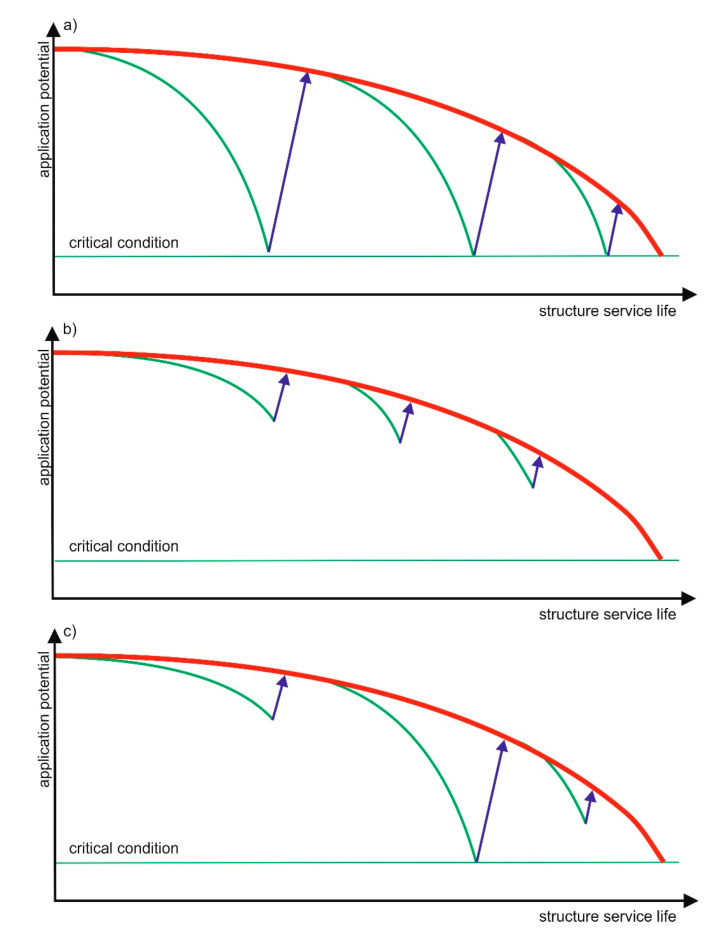
Schematic presentation of repair cycles carried out according to different durability management strategies [28,29]: (**a**) reactive model, (**b**) proactive model and (**c**) combined method red—designed wavelength of the use potential; green—actual wavelength of the use potential; blue—making the repairs.

Generally, there are also more complex structure durability management strategies to be considered [29,30]. Structure durability management methods can be divided into reactive (Figure 6a), proactive (Figure 6b) or mixed (Figure 6c).

In fact, the durability management model can be even more complex, if the applied repair methods impact not only the level of the use potential after the repair but also the degradation rate, stalling it e.g., by applying corrosion inhibitors, or accelerating it e.g., as a result of concentration cell formation following the repair. The latter is normally not intended and, more dangerously, it can go unnoticed. It will be emphasised that structural safety is the most important requirement, which we want to meet as a result of the repair.

## 4. Repair Durability Determinants

Following earlier deliberations, it was demonstrated that the commonly used term “concrete repair durability” is correctly understood as a structure’s durability (service life) after the repair. The subject literature is vast; there are many publications including the term “repair durability” in their titles [31,32,33,34,35,36,37,38,39,40,41].

**Figure 7:**
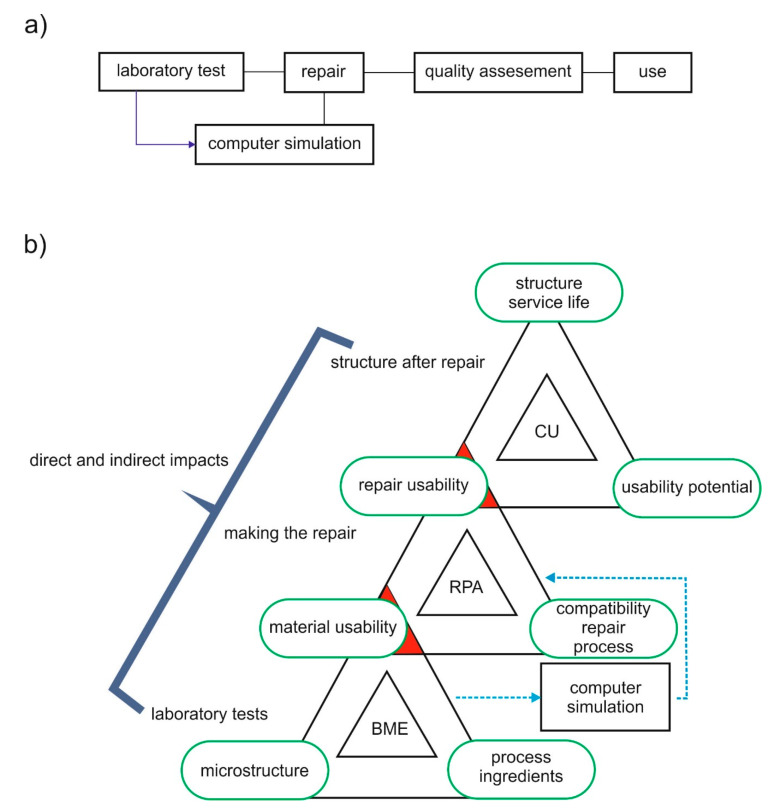
Multi-scale engineering: (**a**) basic operation, (**b**) the repaired structure (based on Mo Li [29]): IMB—Building Materials Engineering; RPA—Repair Performance Assessment; CU—Use of the structure.

Rarely (if at all), the documents give a clear answer, e.g., indicating the number of years which determine repair durability, i.e., the period of maintaining the structure’s service life achieved (regained) owing to repair. This results from the fact that if a successful repair restores the service condition prior to repair, the structures last and the designed service life of a structure is still running. The value added by the repair as such is hard to separate. In the case of repairs made in structures in critical condition (see Section 3), the entire period of service life after the repair can be assigned as “repair durability”. Development of structure durability as a result of repair is regarded as a multi-scale process [18,29] carried out on several levels (Figure 7.).

The repair process operations are carried out taking into consideration direct and indirect impacts, namely both functional and environmental loads.

The relevant publications (all including “repair durability” in their titles) present theoretical substantiation [18,21,30,31,32,34,36] and the technical characteristics that the repair measures should meet [33,34,35,37,38,39] or innovative material solutions [41]. They aim to provide understanding on compatibility requirements, namely what characteristics the repair material should meet to ensure effective repair. The recommendations [42] for the general categories are as follows:

- adhesion—high; it is the prerequisite for successful repair: the higher the adhesion, the higher the tolerances for any potential compatibility errors [43]
- shrinkage—low; excessive shrinkage [44] is often the cause of unsuccessful repairs
- tensile strength—high
- fatigue strength—high [19]
- chloride penetration—low
- susceptibility to carbonation—low
- resistance to aggressive environmental impact—high

These are only one-side limited requirements, usually formulated as in equation: ≥ and ≤.

There is a number of requirements which depend on the repaired concrete characteristics. Concrete of different ages can be repaired. Their values are expressed as closed sets, with the highest and lowest values specified ≤ x ≤, e.g.:

- Young’s modulus,
- creep factor,
- coefficient of thermal expansion,
- water absorbability.

**Figure 8:**
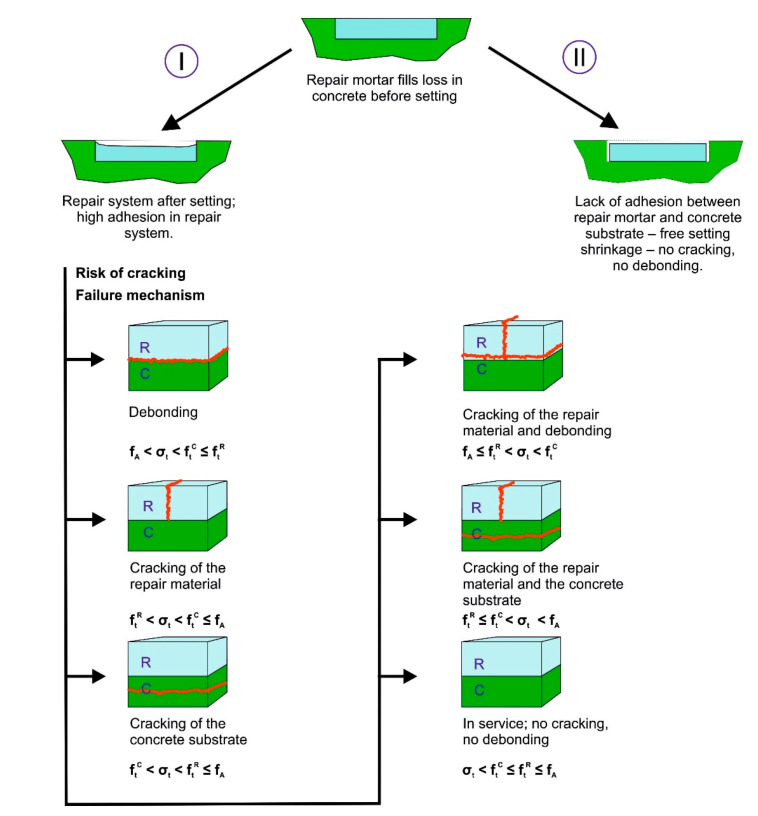
Damage models in repair systems: I—high adhesion in repair system, II—no adhesion, no cracking. Various kind of cracks in repaired system; σ_t—_internal shrinkage stress, f_t_^R—^repair material tensile trenght, f_t_^C^—concrete tensile strength, f_A_—adhesion strength.

It is not surprising that adhesion is a fundamental issue in construction engineering. This is of particular significance for concrete repair, as in this case the repair materials have to be combined with an existing concrete substrate. The most important factor affecting the durability of the repair is the adhesion between repair material and concrete substrate. Approximately, successful repair means “no cracks status”. Considering the damage models and failure mechanisms, the repair system consists of two basic lines (Figure 8: I and II). Paradoxically, if the adhesion in the repaired system would be zero, then the free shrinkage stress setting will be possible and in consequence zero stress result [45]. From a practical point of view, the “zero adhesion repair method” seems to be close to an absurd [46].

The analysis of all cracking models (Figure 8) will show that only when adhesion strength, f_A_ is higher than repair material tensile strength f_t_^R^, and that of concrete tensile strength, f_t_^C^ and also higher than internal shrinkage stress, σ_t_, the status “no cracking” could be achieved. Moreover, in order to ensure the repair durability, the conjuncture f_A_ ≥ f_t_^R^ ≥ f_t_^C^ ≥ σ_t_ should be maintained throughout the expected service life. The maximum bond strength is a basic recommendation for various repair joints. This is also the main indication of the EN 1504-4 repair standard. The bond strength should be above the repaired concrete tensile strength. The level of adhesion ensures the utmost load capacity of the repaired system. The adhesion of the repair joint is effective if it enables load transfer and ensures even distribution of stresses [42].

## 5. Estimating Repair Durability

**Figure 9:**
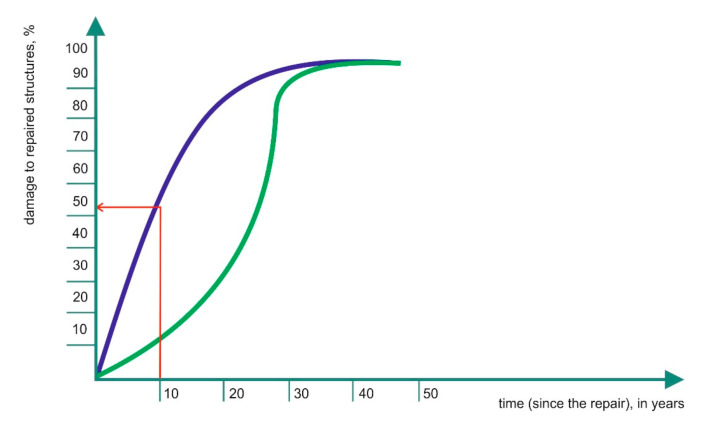
Damage to the repaired structures (repair durability) incrementally in time (navy line); data according to [20]. The green line stands for the simulated distribution for repairs made according to EN 1504-1 ÷ 10.

“The Durability of Repaired Concrete Structure” [21] by G. Tilly from the English company Gifford & Partners, assessing the condition of buildings, is to the best of our knowledge the only work published in the last 30 years devoted to the assessment of the durability of repaired structures (service life), expressed by the number of years “lived” after the repair. A report was published in 2007 as a result of a European project *CONREPNET: Performance-based approach to the remediation of reinforced concrete structures: Achieving durable repaired structures* (EP77 and EP79) [42,47,48]. The assessment covered 230 repaired concrete structures in 24 European countries, from Finland (north) to Greece (south), used in different climate conditions and in urban, industrial, rural and seaside environments. The mean age of the structures ranged from 20–50 years, and the oldest building structure was 150 years old (a church built in 1852). The assessment covered repaired buildings (77), bridges (75), dams (30), car parks (8) and power plants (12). Industrial facilities and tunnels formed the rest. The repairs were forced by corrosion, including freeze corrosion (65%), construction faults (20%), scratches/cracks (10%) and alkaline reaction of aggregates (5%). The highest observed repair durability amounted to 52 years. Based on G. Tilly’s data, it is possible to reconstruct the distribution function of the repaired structural damage over time (Figure 9).

As much as 20% of the repaired structures were damaged within the initial five years after the repair. This means that repair durability in this group did not exceed five years. A total of 55% of the repaired structures were damaged within the initial ten years following their repair. Only 10% exhibited repair durability over 25 years, which should be considered as the desired expected value. In this context, the results are disappointing. It needs to be emphasised though that the data refer to the past. The set of repairs included in G. Tilly’s analysis covers repairs carried out before the implementation of the European standard on repairs EN 1504-1 ÷ 10 (2004–2013). Significant progress was made both in the development of the theoretical fundamentals of repairs and in reference to the repair products and systems. In our opinion, the following rule can be formulated: “if a structure is repaired and protected correctly, according to EN 1504-1 ÷ 10, and if it survives the first year of its use with no reservations, it will last for 10 years”. In the final wording: “if a structure is repaired and protected correctly, according to EN 1504-1 ÷ 10, and if it survives the first year of its use with no reservations, it can last for up to 25 years”. Future experiences will verify the predictions.

**Figure 10:**
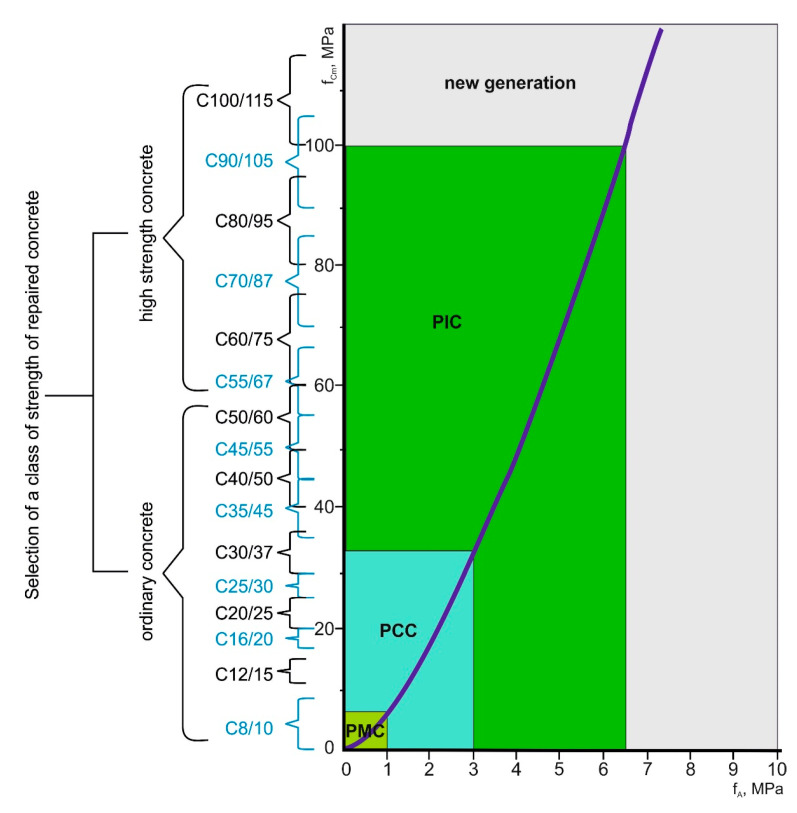
An algorithm of a selection of a polymer repair material depends on class of strength of repaired concrete substrate: PMC—Polymer Modified Concrete (*p* ≤ 1%), PCC—Polymer Cement Concrete (1% < *p* ≤ 3%), PIC—Polymer Concrete (*p* > 8%)—no Portland cement; *p*—polymer content, %—mass of concrete.

In G. Tilly’s paper, repair success is differentiated depending on the applied repair method. For the most common method of defect filling (manually or by spraying), the success factor was only 30%, but if the defect filling was combined with the application of surface protective coating, the success factor increased to 50%. An optimistic projection is the successful restoration of durability (load bearing capacity) by 75% and crack injection by 70%. This is particularly important because crack injection repair cannot be repeated. The main reasons for unsuccessful repairs include: scratches (30%), loosening (25%), progressive corrosion (20%), leakage (7%), aggregate alkaline reaction (3%) and other (15%). The ranking of the reasons emphasises the significance of careful repair and stressed the difficulty related to delaying (interrupting) the inevitable corrosion processes. The adhesion of ordinary fresh concrete to old one is rather weak. This phenomenon is the main reason for the presence of polymer in repair materials as a response to the demand to assure a high level of adhesion (Figure 10).

In general: f_A_ > α f_t_^C^; where α = 1.15 to1.30. The safety factor depends on the various repair materials and different systems; with and without coating [42,49].

At the same time, along with the increase in adhesion, the tolerance to incompatibility of the properties of the joined materials also increases [50,51]. Even the thermal compatibility according to the EN 14043 standard is expressed by adhesion. The higher the adhesion, the higher the tolerance for compatibility error. It should be emphasized that till now it was not elaborated any complex measure of the repair compatibility [23]. A compromise is needed between reliability and durability as well as compatibility and economy.

## 6. Conclusions

In engineering terms, Concrete Repair Durability, CRD, means additional construction service lifetime obtained through successful repairs. This means that CRD should be expressed in time units. In this case, it was necessary to analyse past repair experiences.

The following conclusions have been formulated as a result of the juxtaposition of the modern state of concrete repair technology with selected historical information on concrete repairs and masonry structures repairs.

1. A successful repair means that there are “no cracks” occurring throughout the estimated service life. This means that the adhesion, f_A_ in the repair joints should be above tensile strength of the concrete, f_t_^C^ and internal shrinkage stress, σ_t_. Therefore, we arrive at: f_A_ ≥ f_t_^R^ ≥ f_t_^C^ ≥ σ_t_ that should be maintained throughout the expected service life.
2. The adhesion between repair materials and concrete structures is a decisive factor of radical concrete repairs. The answer to ensuring a high level of adhesion is the presence of the polymer in repair materials. This means that if a repaired concrete is of a higher class, we would need a repair material with a higher polymer content.
3. Concrete repairing impact on the repair durability is acting according to the two mechanisms:
  - Restore the original (or even improved) application potential,
  and/or
  - The reduction of the rate of destruction process.
4. The distribution function of the aforementioned repaired structural damages indicates that the durability of 50% of these repairs is less than 10 years. These poor results relate to historical structures and past attempts at repairing them. The expected repair durability median value is 25 years. Successful repairs are not only dependent on observing best practices in a given period, but also on the skills of people executing the repairs. There is an opportunity to manage repair strategy in a proactive way to assure expected durability of repaired concrete.
